# Compound heterozygous mutations in the TH gene in a Chinese family with autosomal-recessive dopa-responsive dystonia: A case report

**DOI:** 10.1097/MD.0000000000012870

**Published:** 2018-11-02

**Authors:** Bangzhe Feng, Guangfei Sun, Qingxia Kong, Qiubo Li

**Affiliations:** a Cheeloo College of Medicine, Shandong University, Jinan, Shandong; b Department of Pediatrics, Affiliated Hospital of Jining Medical University, Jining, China.

**Keywords:** dopa-responsive dystonia, gene mutation, tyrosine hydroxylase deficiency

## Abstract

**Rationale:**

Autosomal-recessive dopa-responsive dystonia (DRD) is a rare clinical disorder presenting as bradykinesia, dystonia, tremor and even severe encephalopathy, and caused by tyrosine hydroxylase deficiency (THD). We report a case of compound heterozygous mutations in the TH gene in a Chinese family with autosomal-recessive DRD herein.

**Patient concerns:**

A 16-month-old Chinese boy presented with symptoms of movement disorder and growth retardation in his infant period.

**Diagnoses:**

The genetic test revealed compound heterozygous mutations in the TH gene at c.457C>T and c.698G>A, which are pathogenic of DRD.

**Interventions:**

The patient was administrated low-dose levodopa.

**Outcomes:**

The treatment resulted in the substantial improvement of dystonia. His long-term neurological outcome need follow-up for years.

**Lessons:**

Gene mutation analysis is helpful and necessary to diagnose DRD and has important guiding significance for the subsequent treatment.

## 1 Introduction

Dopa-responsive dystonia (DRD), also known as Segawa syndrome, is a hereditary dyskinetic disorder. Autosomal-dominant DRD is a much more common neurotransmitter disorder in the population due to mutations in the GTP cyclohydrolase 1 (GCH1) gene on chromosome 14q22.1–22.2 compared to the autosomal-recessive DRD due to mutations in the tyrosine hydroxylase (TH) gene on chromosome 11p15.5.^[1,2]^ The enzyme TH, a key enzyme in the biosynthesis of the catecholamines dopamine, epinephrine and norepinephrine, catalyzes the conversion of L-tyrosine to L-dihydroxyphenylalanine (L-DOPA).^[3]^ If mutations occur in the TH gene, it will directly affect the level of catecholamine and its downstream products such as homovanillic acid (HVA) and 3-methoxy-4-hydroxyphenylethylene glycol (MHPG), which are meaningful to diagnosis, causing a series of clinical symptoms such as hypokinesia, bradykinesia, rigidity, dystonia, chorea, tremor, oculogyric crises, ptosis, hypersalivation and other symptoms due to cerebral dopamine and norepinephrine deficiency.^[4]^

## 2 Methods

Genomic DNA was extracted from the peripheral blood of the subject in order to construct genomic libraries. The exon region and adjacent intron region of the entire human gene were hybridized using the exon trapping kit SureSelectXT Human All Exon V6 (Agilent, California) after processes of fragmentation, amplification and purification. The captured DNA was processed with elution, amplification and purification and sequenced at a high-throughput sequencer (Illumina, California). Sequencing data were compared with human reference genome sequence UCSC. hg19 with NextGene V2.3.4 software (SoftGenetics, Pennsylvania) to identify the genetic variations.

Sanger sequencing was performed on the mutation region of the child. Forward primer of exon 4 in the TH gene: 5′- TGCTGCTAGCACAAAAGTCAAGG-3′; reverse primer: 5′- CGGACACGGATGTGTAGCAAAAC-3′. The length of the amplified product was 468 bp. Forward primer of exon 6 in the TH gene: 5′- TCTTGTAGGGGAGGCTGCTTC-3′; reverse primer: 5′- GCACTGATGCTGGTGACAAGATG-3′. The length of the amplified product was 513 bp. The PCR reaction volume was 25 μL: 2.5 μL of 10 × Taq buffer, 2.0 μL of dNTPs (2.5 mM), 0.25 μL of Taq polymerase, 50 ng of DNA template, 0.5 μL of each of the upstream and downstream primers (5 μmol/L), and deionized water was added to 25 μL. PCR amplification: pre-denaturation at 95°C for 5 minutes, denaturation at 95°C for 30 seconds, annealing at 60°C for 30 seconds, extension at 72°C for 40 seconds, 35 cycles, and finally extension for 5 minutes. The results were obtained by using the gel imaging analysis system.

## 3 Case report

A 16-month-old boy presented with growth retardation and hypotonia. He was the second child who was born to non-consanguineous Chinese couple. His elder sister developed normally. He had an uncle and a cousin on his mother's side. His parents claimed that their relatives had no similar medical history.

This child was born 5 weeks prematurely by cesarean section. His birth weight was 2.0 kg with no history of asphyxia. He often experienced the symptoms of coughing, snoring and stuffy nose after birth. The first 3 months after birth, he developed relatively normally. Nevertheless, it was found that his independent activities were less than his peers when he was 3 months old. After that, he began to present with crying weakness, limb weakness and hypotonia, accompanied by diurnal symptom marked fluctuation. Then he was diagnosed as “growth retardation” and suspected as “cerebral palsy”. Two months later, he still had a poor strength to grip. He could not suck his finger or look up more than 45 degrees in the prone position. He was also unable to keep his neck stable vertically when he was pulled up. He could recognize parents and understand the meaning of their talk. There were no remarkable symptoms such as abnormal eye movements, convulsion, hidrosis, and ptyalism.

There were no obvious peripheral nerve abnormalities in electromyography (EMG) examination. Brain magnetic resonance imaging showed bilateral widened frontotemporal extracerebral space which in line with imaging manifestations of premature children. After physical examination and neostigmine test, the possibility of myasthenia gravis (MG) was ruled out. It was suspected that he may suffer from spinal muscular atrophy (SMA) in children because of myasthenia and dyskinesia, but no relevant genetic pathogenic mutations were detected by molecular genetic study.

He was hospitalized in Beijing at the age of 6 months. It was considered that he suffered from congenital hereditary metabolic disease based on the aforementioned characteristics. His blood cell counts, hemoglobin, electrolytes, C-reactive protein, lipids, blood glucose, liver function tests, serum ceruloplasmin, urine gas chromatography-mass spectrometry, tandem mass spectrometry, and biotinidase were unremarkable. 3-hydroxybutyric acid level elevated which suggested ketonuria. Urine neopterin level was within normal limits and biopterin level was slightly lower. As an end product from adrenaline and norepinephrine, the urine vanillylmandelic acid (VMA) level was significantly decreased [2.46 μmol/24 hours, (reference level:11.7–84.6 μmol/24 hours)].

**Figure 1:**
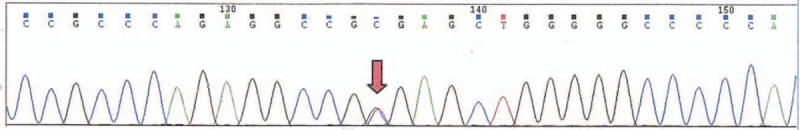
The sequencing chromatograms of proband revealed c.457C>T in the TH gene. TH = tyrosine hydroxylase.

**Figure 2:**
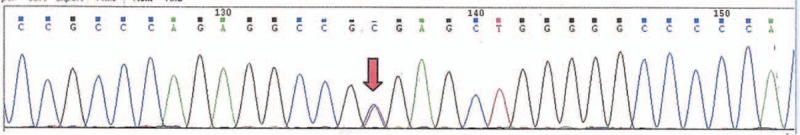
The sequencing chromatograms of the father revealed c.457C>T in the TH gene. TH = tyrosine hydroxylase.

**Figure 3:**
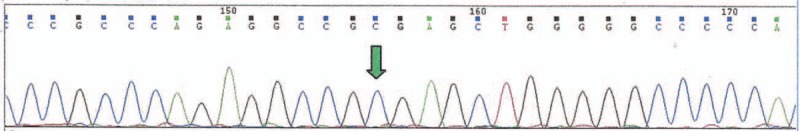
The sequencing chromatograms of the mother found no mutations at the site of 457 in the TH gene. TH = tyrosine hydroxylase.

**Figure 4:**
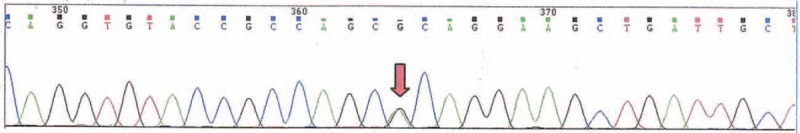
The sequencing chromatograms of proband revealed c.698G>A in the TH gene. TH = tyrosine hydroxylase.

**Figure 5:**
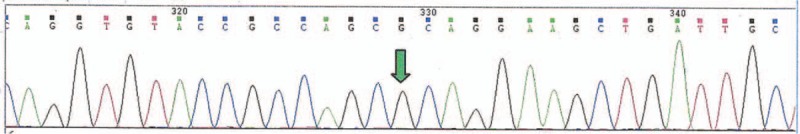
The sequencing chromatograms of the father found no mutations at the site of 698 in the TH gene. TH = tyrosine hydroxylase.

**Figure 6:**
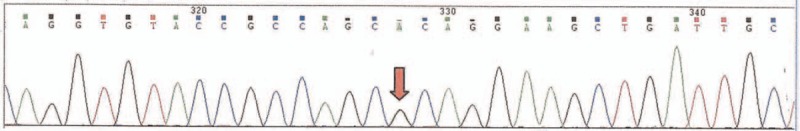
The sequencing chromatograms of the mother revealed c.698G>A in the TH gene. TH = tyrosine hydroxylase.

After obtaining informed consent, the genetic analysis revealed 2 heterozygous mutations c.457C>T (paternal) and c.698G>A (maternal) (Figs. 1 and 4), which resulted in amino acid changes p.R153X and p.R233H. His parents were subjected to genetic tests, and they proved to be healthy carriers afterwards (Figs. 2 and 3, Figs. 5 and 6) while his elder sister was not subjected to the test. This child was diagnosed with DRD and treated with levodopa.

According to our follow-up, his condition improved dramatically after the treatment with 1/12 tablet of a levodopa 200 mg/benserazide 50 mg combination twice daily. The patient adhered to treatment with help from his parents. He weighed 7.5 kg and could raise his head at the age of 11 months. One month later, he was able to turn over and call “mom” and “dad”. Sitting alone can be maintained for about 10 seconds with the armrest when he was 14 months old. The dose was increased to 1/8 tablet, 3 times per day at the age of 15 months. His parents found no obvious problem during the treatment course.

## 4 Discussion

DRD is a hereditary disorder that usually occurs in children and adolescents with marked fluctuation. It is distinguished by a dramatic and sustained effect with treatment of low-dose L-DOPA. Above mentioned mutations in the TH gene were identified as pathogenic mutations of DRD by Human Gene Mutation Database.^[5–8]^ To our knowledge, these mutation sites have rarely been reported in mainland China. This genetic data has not been shared with archive and there is no accession number so far.

The missense mutation in the TH gene and its promoter region is the cause of TH deficiency. Mutations have significant effects on TH solubility, activity, stability and other aspects.^[6]^ In contrast, autosomal-dominant DRD caused by mutations in the GCH1 gene is less severe and characterized by a later onset. At the age of 3 months, the child developed symptoms of limb weakness and hypotonia with diurnal variation, which were coincided with the clinical features of DRD. Based on physical examinations, laboratory examinations and genetic studies, possibilities of MG, pediatric SMA, 6-pyruvoyl-tetrahydropterin synthase deficiency, and other diseases were ruled out successively. Hence, it was diagnosed as DRD due to tyrosine hydroxylase deficiency (THD, OMIM 191290). Clinical phenotypes of THD can be divided into type A and type B. The former is manifested by infantile hypokinetic-rigid syndrome with dystonia with better response to L-DOPA. The latter is characterized by neonatal complex encephalopathy with poor response to the treatment of L-DOPA in most cases.^[9]^ L-DOPA-induced dyskinesias is more likely to occur in patients with type B THD.^[10]^ Our patient is classified as type A THD. The TH knock-in mouse with p.Arg203His mutation reconfirms the human type B THD phenotype including biochemical markers, hypokinesia, diurnal symptom fluctuation and poor response to L-DOPA by experiment. Manifestations of THD are quite similar to many neurological disorders. it is not easy to make an accurate diagnosis because of its rarity and features. This disease is rarely reported in mainland China, to some extent, correlated with a lack of clinicians’ cognition of relevant knowledge. So that is the reason why the patient was not diagnosed correctly and quickly at the beginning. Unfortunately, the genetic analysis was not performed for his elder sister for some reason. Furthermore, his long-term neurological outcome still needs follow-up for years. With reasonable adjustments of dosage and scientific rehabilitation training, the patient's condition will be most likely to improve with the time. We hope to provide some reference of the treatment of autosomal-recessive DRD through this case, especially for mainland China.

At present, normal cerebrospinal fluid (CSF) concentrations of 5-hydroxyindole acetic acid (5-HIAA) with decreased HVA and MHPG. CSF levels are recognized as important biochemical markers of THD.^[9]^ Some patients are reluctant to have the invasive tests, so genetic analysis is 1 of the most significant means for diagnosis of THD. Dopamine is the main inhibitory factor of prolactin and therefore the level of prolactin probably increase in THD patients.^[11]^ In addition, the CSF synaptic proteins dopamine transporter, D2-receptor and vesicular monoamine transporter are regarded as useful biomarkers for THD.^[12]^

The effective treatment strategy for THD is relatively clear. Previous cases have shown successful improvements among most patients with THD who are treated with levodopa/carbidopa, but the curative effect varies with different individuals. Deep brain stimulation of bilateral subthalamic nucleus which is helpful to alleviate dystonia and reduce demand for drugs is an interesting exploration as well.^[13]^ If our patient's parents want to have another child, the probability of fetus involvement in THD is 25%. Therefore, early diagnosis and intervention, strengthening publicity and education for doctors at all levels are of great significance to treat this kind of diseases and reduce the burden on patients and their families.

## 5 Conclusion

In conclusion, DRD should be considered for unexplained dystonia in infancy, especially with extrapyramidal symptoms. In our case, the patient was detected with heterozygous mutations at c.457C>T and c.698G>A in the TH gene. Genetic testing is the most important diagnostic method and also has guiding significance for follow-up treatment.

## Author contributions

**Data curation:** Bangzhe Feng.

**Formal analysis:** Bangzhe Feng.

**Investigation:** Bangzhe Feng, Guangfei Sun.

**Resources:** Qingxia Kong, Qiubo Li.

**Supervision:** Qingxia Kong, Qiubo Li.

**Writing – original draft:** Bangzhe Feng.

**Writing – review & editing:** Guangfei Sun, Qingxia Kong, Qiubo Li.
